# Aneuploidy related transcriptional changes in endometrial cancer link low expression of chromosome 15q genes to poor survival

**DOI:** 10.18632/oncotarget.14201

**Published:** 2016-12-25

**Authors:** Karen Klepsland Mauland, Elisabeth Wik, Erling A. Hoivik, Kanthida Kusonmano, Mari Kyllesø Halle, Anna Berg, Hans Kristian Haugland, Anne Øyan Margrete, Karl-Henning Kalland, Ingunn Marie Stefansson, Lars A. Akslen, Camilla Krakstad, Jone Trovik, Henrica Maria Werner Johanna, Helga Birgitte Salvesen

**Affiliations:** ^1^ Center for Cancer Biomarkers CCBIO, Department of Clinical Science (K2), University of Bergen, Bergen, Norway; ^2^ Department of Obstetrics and Gynecology, Haukeland University Hospital, Bergen, Norway; ^3^ Center for Cancer Biomarkers CCBIO, Department of Clinical Medicine (K1), Section for Pathology, University of Bergen, Bergen, Norway; ^4^ Department of Pathology, Haukeland University Hospital, Bergen, Norway; ^5^ Computational Biology Unit, University of Bergen, Bergen, Norway; ^6^ Bioinformatics and Systems Biology Program, School of Bioresources and Technology, King Mongkut's University of Technology Thonburi, Bangkhuntien, Bangkok, Thailand; ^7^ Department of Microbiology, Haukeland University Hospital, Bergen, Norway; ^8^ Center for Cancer Biomarkers CCBIO, Department of Biomedicine, University of Bergen, Bergen, Norway

**Keywords:** endometrial cancer, DNA ploidy, prognosis, transcriptional alterations, chromosome 15q

## Abstract

Aneuploidy is a widely studied prognostic marker in endometrial cancer (EC), however, not implemented in clinical decision-making. It lacks validation in large prospective patient cohorts adjusted for currently standard applied prognostic markers, including estrogen/progesterone receptor status (ER/PR). Also, little is known about aneuploidy-related transcriptional alterations, relevant for understanding its role in EC biology, and as therapeutic target.

We included 825 EC patients with available ploidy status and comprehensive clinicopathologic characterization to analyze ploidy as a prognostic marker. For 144 patients, gene expression data were available to explore aneuploidy-related transcriptional alterations.

Aneuploidy was associated with high age, FIGO stage and grade, non-endometrioid histology, ER/PR negativity, and poor survival (p-values<0.001). In patients with ER/PR negative tumors, aneuploidy independently predicted poor survival (p=0.03), lymph node metastasis (p=0.007) and recurrence (p=0.002). A prognostic ‘aneuploidy signature’, linked to low expression of chromosome 15q genes, was identified and validated in TCGA data.

In conclusion, aneuploidy adds prognostic information in ER/PR negative EC, identifying high-risk patients that could benefit from more aggressive therapies. The ‘aneuploidy signature’ equally identifies these aggressive tumors and suggests a link between aneuploidy and low expression of 15q genes. Integrated analyses point at various dysregulated pathways in aneuploid EC, underlining a complex biology.

## INTRODUCTION

Aneuploidy, defined as an aberrant number of chromosomes, is a commonly observed feature in human cancers [1], including endometrial cancer (EC) [2]. Aneuploidy is often evaluated in tumors as an indirect measure of chromosomal instability, and used as an indicator of poor outcome [3]. It has been among the most widely studied biomarkers in EC since it was first introduced in the eighties [4–7], and is of particular interest since it can be measured preoperatively and hence used to guide treatment decisions [8–10]. Ploidy status estimated by cytometric methods [11] has added prognostic information in several retrospective studies of EC, but has never been fully implemented in clinical treatment algorithms [12]. This is at least in part due to the lack of one common standardized method for measuring ploidy status in tumors [3]. In addition, its clinical usefulness as prognostic marker, adjusted for clinical and histopathologic variables, lacks validation in large prospective patient cohorts [13].

Aneuploidy is suggested to arise through a few major mechanisms, including mitotic checkpoint defects, centrosome over-duplication and defect sister chromatid cohesion [14], for instance by mutational loss of the cohesin subunit *STAG2* [15]. However, the exact role of aneuploidy in tumor development and progression remains incompletely understood, and no single causative driver has been identified [16]. For EC, the prognostic impact of aneuploidy has been studied to a large extent, but associated transcriptional alterations have been much less explored. This is relevant for identifying shared molecular traits among aneuploid endometrial tumors, and hence to understand more about underlying biologic mechanisms in aggressive EC with possible relevance for new targeted therapies.

We therefore evaluated flow cytometry assessed DNA ploidy status in a large well-annotated EC patient cohort with long and complete follow-up, and demonstrated clear associations between aneuploidy and markers of poor outcome. Further, we examined transcriptional alterations reflecting ploidy status in primary EC lesions, revealing a prognostic ‘aneuploidy signature’ linked to low expression of chromosome 15q genes, and shedding light on biologic mechanisms accompanying aneuploidy in EC.

## RESULTS

### Aneuploidy associates with markers for aggressive endometrial cancer

**Figure 1:**
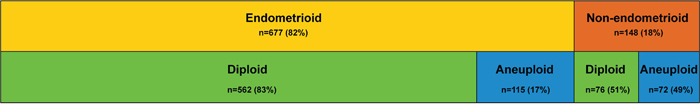
Ploidy status in histologic subtypes of endometrial cancer Schematic overview of ploidy status (by flow cytometry) according to histologic subtype for 825 patients. n=number of patients in each category (percent).

Of the 825 tumor samples with flow cytometry estimated ploidy status available, 638 were diploid (77%) and 187 aneuploid (23%). Example DNA histograms are shown in Supplementary Figure S1. Aneuploidy was significantly associated with well-established prognostic variables, including high age, FIGO stage and grade, non-endometrioid histology, and estrogen receptor and progesterone receptor (ER/PR) negativity (Table 1). The proportion of diploid and aneuploid tumors according to histologic subtype is shown in Figure 1. The frequency of aneuploid tumors was 38% among patients who later suffered recurrence and 42% in patients with metastasis at primary diagnosis, compared to 17% for patients without signs of systemic or recurrent disease (p<0.001).

**Table 1:** Associations between clinicopathologic factors and DNA ploidy status by flow cytometry in 825 endometrial carcinomas

	Diploid, n (%)	Aneuploid, n (%)	p-value*
**Age, quartiles**			
<58	178 (91)	18 (9)	<0.001
58 – 66	171 (83)	35 (17)	
66 – 75	145 (69)	66 (31)	
≥ 75	144 (68)	68 (32)	
**Histologic type and grade^a^**			<0.001
Endometrioid, grade 1-2	467 (87)	71 (13)	
Endometrioid, grade 3	75 (63)	44 (37)	
Non-endometrioid	76 (51)	72 (49)	
**FIGO stage**			<0.001
Stage I	502 (81)	121 (19)	
Stage II	54 (74)	19 (26)	
Stage III	62 (66)	32 (34)	
Stage IV	20 (57)	15 (43)	
**ER/PR status^b^**			<0.001
ER and/or PR positive	389 (82)	84 (18)	
ER and PR negative	72 (61)	46 (39)	

n=number of patients in each category; *: Pearson χ^2^-test; ^a^: Data missing for 20 patients; ^b^: Data missing for 234 patients

### Aneuploidy associates with reduced survival

**Figure 2:**
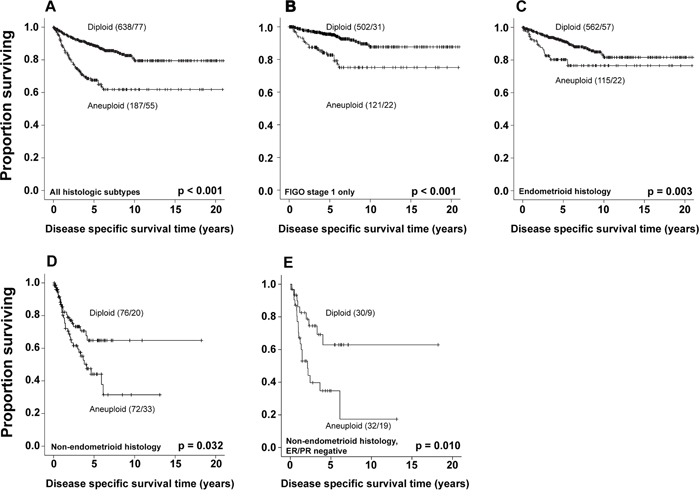
Survival according to ploidy status Kaplan-Meier curves showing DSS according to ploidy status, for all histologic subtypes **A**., FIGO stage 1 tumors **B**., endometrioid histologic type **C**. non-endometrioid histologic type **D**., and ER/PR negative non-endometrioid histologic type **E**. For each category the number of cases is given, followed by number of deaths.

In univariate survival analysis (Kaplan-Meier), the 5-year disease specific survival (DSS) for patients with diploid tumors was 89%, versus 68% for patients with aneuploid tumors (p<0.001, Figure 2A). Subgroup analyses confirmed aneuploidy as a significant marker for shorter survival in patients with FIGO stage 1 (Figure 2B), as well as endometrioid and non-endometrioid tumors separately (Figure 2C and 2D). We also explored to what extent ploidy adds prognostic information to endometrioid and non-endometrioid subgroups with known ER/PR status. In non-endometrioid tumors with positive ER/PR status (n=52), ploidy did not show any prognostic impact in univariate analysis (p=0.800, data not shown). However, ploidy significantly affected outcome in non-endometrioid hormone receptor negative tumors (n=62), with 5-year DSS of 63% for patients with diploid versus 35% for aneuploid tumors (p=0.010, Figure 2D).

Lymphadenectomy is advocated as staging procedure, and is documented to improve prognostication, however without effect on survival in randomized trials [17, 18]. We therefore explored the prognostic impact of ploidy compared to ER/PR for three subgroups: patients with lymph node metastases, without lymph node metastases, and where lymphadenectomy was not performed. For patients with lymph node metastasis, ER/PR performed better than ploidy status (Supplementary Figure S2A and S2B). In the other two groups, ER/PR status and ploidy added similar prognostic information in univariate survival analyses (Supplementary Figure S2C-S2F).

In multivariate analysis, ploidy maintained independent prognostic impact on DSS, adjusting for the commonly applied standard prognostic markers: age, FIGO stage, histologic subtype and grade; with a hazard ratio (HR) of 1.62 for aneuploid tumors (95% confidence interval (CI) 1.11 – 2.37, p=0.013 (Supplementary Table S1A). Due to a detected significant interaction between ER/PR and ploidy status (HR 3.03, 95% CI 1.23 – 7.42, p=0.016), the two variables were not included simultaneously in the multivariate model. Replacing ploidy with ER/PR status in the Cox model, a similar HR of 1.63 (95% CI 1.16 – 2.29, p=0.005) for ER/PR negativity was observed (Supplementary Table S1B). In subsequent Cox analyses stratified for ER/PR status, aneuploidy independently predicted poor outcome in the receptor negative group only, with HR 2.11 (95% CI 1.08 – 4.15, p=0.029) (Table 2A and 2B).

**Table 2:** Prognostic impact of ploidy status by flow cytometry adjusted for standard clinicopathologic variables, stratified for ER/PR expression (Cox regression model)

	Paitents, n (%)	Unadjusted HR	95% CI	p-value	Adjusted HR	95% CI	p-value
**Age**	470 (100)	1.05	1.03 - 1.08	<0.001	1.04	1.02 - 1.07	0.001
**Histologic type and grade**				<0.001			<0.001
Endometrioid grade 1-2	356 (76)						
Endometrioid grade 3	62 (13)	4.57	2.41 - 8.66		2.81	1.44 - 5.47	
Non-endometrioid	52 (11)	9.08	4.75 - 17.34		4.51	2.16 - 9.43	
**FIGO stage**				<0.001			<0.001
Stage I – II	409 (87)						
Stage III – IV	61 (13)	11.53	6.80 - 19.56		8.97	5.12 - 15.70	
**Ploidy status**				0.053			0.666
Diploid	386 (82)						
Aneuploid	84 (18)	1.80	0.99 - 3.24		0.87	0.45 - 1.66	
	**Patients, n (%)**	**Unadjusted HR**	**95% CI**	**p-value**	**Adjusted HR**	**95% CI**	**p-value**
**Age**	117 (100)	1.04	1.01 - 1.08	0.013	1.04	1.00 - 1.08	0.082
**Histologic type and grade**				0.009			0.184
Endometrioid grade 1-2	27 (23)						
Endometrioid grade 3	28 (24)	0.87	0.27 - 2.85		1.02	0.31 - 3.41	
Non-endometrioid	62 (53)	2.82	1.16 - 6.84		1.99	0.81 - 4.90	
**FIGO stage**				<0.001			<0.001
Stage I – II	78 (67)						
Stage III – IV	39 (33)	6.66	3.39 - 13.07		5.56	2.77 - 11.13	
**Ploidy status**				0.001			0.029
Diploid	71 (61)						
Aneuploid	46 (39)	3.06	1.61 - 5.82		2.11	1.08 - 4.15	

n=number of patients; HR: Hazard Ratio; CI: Confidence interval

### Aneuploidy independently predicts lymph node metastasis and recurrence

Ploidy status was analyzed for its ability to predict lymph node metastasis and recurrence in binary logistic regression models adjusting for histologic subtype and grade. Due to the previously detected interaction, analyses were stratified for ER/PR status. In the group with ER/PR negative status, aneuploidy independently predicted recurrence (n=96), with odds ratio (OR) 4.67 (95% CI 1.78 – 12.27, p=0.002; Supplementary Table S2A), and lymph node metastasis (n=76) with OR 5.47 (95% CI 1.58 – 18.99, p=0.007, Supplementary Table S2B). Thus, ploidy assessment may be a useful additional biomarker especially in ER/PR negative tumors, identifying high-risk patients that could benefit from further systemic treatment.

### Aneuploidy and its phenotype is reflected in a nine-gene ‘aneuploidy signature’

**Figure 3:**
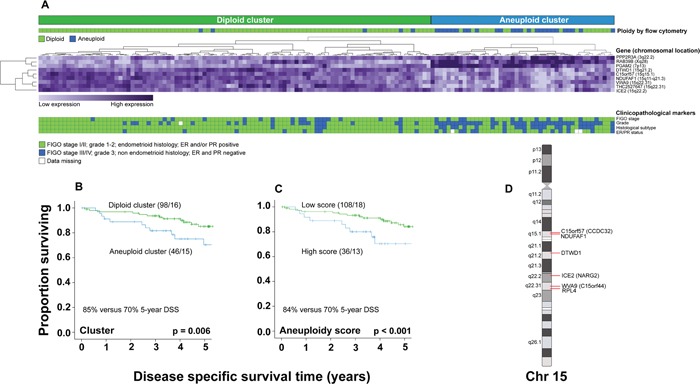
‘Aneuploidy signature’ Formation of diploid and aneuploid clusters based on the ‘aneuploidy signature’, related to flow cytometry ploidy status, FIGO stage, grade, histologic subtype, and ER/PR status. Unsupervised hierarchical clustering of 144 samples **A**. DSS for patients segregating within the ‘diploid’ compared to the ‘aneuploid cluster’ **B**., and for patients with low versus high ‘aneuploidy scores’ **C**. Schematic mapping of the six downregulated ‘aneuploidy signature’ genes, located on chromosome 15q **D**.

Significance analysis of microarray (SAM) was applied to assess aneuploidy related transcriptional alterations. Further, the machine learning algorithm support vector machine (SVM) was applied on the ranked SAM-list in order to identify the genes best discriminating between diploid and aneuploid samples. The nine top ranked genes from SAM were identified as the best discriminators. Of these, six were down- and three were upregulated (Supplementary Table S3). Unsupervised hierarchical clustering of the nine genes identified two patient clusters (Figure 3A). As expected, the ‘aneuploid cluster’ (n=46) reflected more aggressive clinical behavior, comprising the majority (83%) of flow cytometry aneuploid tumors, whereas the ‘diploid cluster’ (n=98) reflected less aggressive clinical features, dominated by flow cytometry diploid tumors (95%). Patients in the ‘aneuploid cluster’ had significantly worse survival than patients in the ‘diploid cluster’ (p=0.006, Figure 3B). An ‘aneuploidy score’ was calculated from expression values of the nine genes [19]. Similar to flow cytometry-assessed aneuploidy, a high score was significantly associated with all markers of aggressive disease (Supplementary Table S4), and reduced survival (p<0.001, Figure 3C). Patients with flow cytometry aneuploid tumors had higher ‘aneuploidy scores’ than patients with flow cytometry diploid tumors (p<0.001, Supplementary Figure S3A). Interestingly, patients with flow-cytometry diploid/gene cluster aneuploid tumors had significantly higher ‘aneuploidy scores’ than patients with flow cytometry-assessed aneuploid/gene cluster diploid tumors (p<0.001, Supplementary Figure S3B). The score increased from premalignant through low grade malignant to high-grade malignant lesions. However, it did not increase further in metastatic lesions (Supplementary Figure S3C).

To further validate the link between the ‘aneuploidy score’, tumor aneuploidy and survival, the score was calculated from TCGA RNAseq data for 338 EC patients with ploidy status estimated by the ABSOLUTE algorithm [20]. We found a similar pattern for cluster formation based on the nine genes: patients segregated into two distinct clusters, one ‘aneuploid cluster’ including 41% of the tumors, associated with a more aggressive phenotype (high FIGO stage, p=0.04, high histologic grade and high stage, both p<0.001), and one ‘diploid cluster’ including tumors with less aggressive features. High ‘aneuploidy score’ also reflected reduced survival in this validation series (Supplementary Figure S4). The larger proportion of tumors in the TCGA ‘aneuploid cluster’ compared to our data set may reflect the TCGA strategy to enrich for more aggressive subtypes in the endometrial cancer series.

Since the proportion of aneuploid tumors differs significantly between histologic subtypes (Figure 1), we also explored the ‘aneuploidy signature’ in endometrioid and non-endometrioid tumors separately. The results for the endometrioid group were similar to those of the whole series, but more difficult to interpret in the non-endometrioid group with small sample size (n=28) (Supplementary Figure S5A-S5B).

### Aneuploidy is related to low expression of genes on chromosome 15q

Interestingly, all six downregulated genes in the ‘aneuploidy signature’ (i.e. low expression in aneuploid compared to diploid samples) were located on the q-arm of chromosome 15 (Figure 3D, Supplementary Table S3). To explore whether loss of 15q regions is a general feature of endometrial cancers, we assessed copy number data for endometrial cancer by the TCGA copy number portal [21, 22], without accounting for ploidy status. 79 peak regions of deletion were identified, of which three located to chromosome 15q. Two of these regions included four of the aneuploidy signature genes: *C15orf57* and *NDUFAF1* in one peak region (residual q-value 3.44 × 10^-5^) and *WVA9* and *RPL4* in another peak region (residual q-value 0.0057). Further, we investigated the publicly accessible cBioportal for copy number alterations of the six genes in relation to mRNA expression level. For all six genes, a proportion of patients had deletion, with correspondingly lower mRNA expression levels compared to patients with normal gene copy number (Supplementary Figure S6). Gene Set Enrichment Analysis (GSEA) with MSigDB c1 positional gene sets, where each gene set corresponds to a cytoband on a human chromosome, showed that nine of the top 30 gene sets enriched in diploid tumors (FDR<25%), were located on chromosome 15q (Supplementary Table S5A). The same analysis was performed in subgroups of endometrioid and non-endometrioid tumors separately, with consistent results (Supplementary Figure S5C). These findings support the argument that aneuploidy is associated with reduced expression of genes on chromosome 15q, possibly due to deletion of chromosomal regions.

### Integrated analyses of aneuploid tumors suggest the involvement of a variety of biological mechanisms and potential drug targets

GSEA identified gene sets related to a wide range of tumorigenic pathways and processes, including cell cycle regulation, cell proliferation and protein transcription as significantly enriched in aneuploid tumors. Notably, several gene sets related to glycolysis were enriched in aneuploid samples. Also, gene sets related to increased expression of known oncogenes including *E2F, KRAS* and *MYC* were frequently upregulated, as well as gene sets related to pluripotency, telomere maintenance and longevity (Supplementary Table S5B). These results suggest that a variety of biological mechanisms may be important in aneuploid tumors, further supported by connectivity map analysis. The 15 top-ranked compounds with negative enrichment score showed a large diversity of drugs potentially relevant for targeting aneuploid tumors (Supplementary Table S6).

### Investigation of aneuploidy-related biomarker potential by STAG2 and PPP2R3A

Since mutational *STAG2* inactivation may be involved in aneuploidy development [15], we investigated STAG2 protein expression by IHC as a potential marker for aneuploidy. No association was found between nuclear STAG2 expression and ploidy status, other prognostic markers except ER status, or survival (Supplementary Table S7, Supplementary Figure S7A-S7C). Further, since PP2A complex alterations have been linked to whole genome doubling [21] and *PPP2R3A* expression was upregulated in the ‘aneuploidy signature’, we also evaluated PPP2R3A protein expression by IHC as a potential aneuploidy marker. No association between PPP2R3A expression and ploidy status, clinicopathologic variables or survival was observed (Supplementary Table S7, Supplementary Figure S7D-S7F).

## DISCUSSION

Despite extensive support for ploidy as a prognostic factor in endometrial cancer [4–7, 23–25], ploidy assessment is not implemented in routine clinical practice. The independent prognostic impact of aneuploidy, after adjusting for histopathologic parameters and surgical staging, is still uncertain, as all these parameters were often not included in earlier studies [4–7, 23–25]. Likewise, the prognostic impact of hormone receptor status in endometrial tumor tissue is well documented [26–28], and easily assessed by immunohistochemistry, however not applied in the routine diagnostic setting either. Nevertheless, the prognostic information derived from ploidy assessment has rarely been compared to hormone receptor status [29], a gap we have tried to fill. In multivariate survival models, ploidy status and ER/PR status contributed similar independent prognostic information based on adjusted hazard ratios. Interaction between ploidy status and ER/PR status was observed in multivariate survival and binary logistic regression analysis, and importantly, aneuploidy further improved the prediction of prognosis, lymph node metastasis and recurrence in tumors with loss of both receptors, all novel observations not previously reported. To our knowledge, interaction between ploidy status and hormone receptor status has never been reported before, and the biological underpinnings of this interesting phenomenon should be further explored in future studies. Also, in this large patient series with a substantial proportion subjected to staging lymphadenectomy, we demonstrated clear prognostic impact of ploidy status, especially in the group of patients where staging lymphadenectomy was not performed. These findings could have potential clinical impact, especially for identification of high-risk patients with need for further adjuvant therapy and closer follow-up.

There are likely numerous causes and consequences of aneuploidy [30]. We aimed to describe transcriptional traits characterizing aneuploid tumors to further explore mechanisms involved in aneuploidy development and/or maintenance in EC, and potentially reveal drugs of interest for further study. In this context, we are only aware of one differently designed study of 33 EC patients [31]. By GSEA, we identified gene sets related to cell cycle regulation, proliferation and transcription to be important in aneuploid tumors, similar to Habermann *et al.’s* findings using ingenuity pathway analysis. GSEA also identified gene sets related to a range of known oncogenes, including *E2F, MYC* and *KRAS*, as well as gene sets linked to cell longevity and pluripotency. This supports the argument that diverse pathways are dysregulated in aneuploid tumors. Connectivity Map analysis pointed towards a wide variety of drugs rather than one specific drug class, again supporting a complex biology underlying aneuploidy in EC, that might require diverse targeting approaches.

Previous attempts to characterize aneuploidy related transcriptional alterations in preclinical models have identified genes related to cellular stress response, response to reactive oxygen species and activated glycolysis as commonly upregulated in aneuploid cells [32, 33]. In line with this, gene sets related to glycolysis were identified as enriched in aneuploid tumors by GSEA. The upregulated ‘aneuploidy signature’ gene *PGAM2* (phosphoglycerate mutase 2), encodes a glycolytic enzyme whose activity increases in response to oxidative stress [34]. Increased *PGAM2* level may indicate a link between oxidative stress, increased glycolysis and aneuploidy in EC, although this association needs to be further elucidated. Also, in a study of colorectal cancer [35], a cancer type sharing several molecular traits with EC [36–38], copy number alterations with correlated expression changes (including 15q loss), affected the activity of the oxidative phosphorylation pathway [35]. Thus, a shift towards anaerobic glycolysis seems tentatively linked to aneuploidy across comparable tumor types. This should be further explored in particular for the development of new therapeutics.

*PPP2R3A*, encoding a subunit of the protein phosphatase 2A (PP2A) complex [39], was also upregulated in the ‘aneuploidy signature’, although we found no association between PPP2R3A expression level by IHC and ploidy status. PP2A complex subunits seem to be frequent targets in EC. *PPP2R1A* mutations often co-occur with *TP53* mutations, a marker for the serous-like subgroup with increased copy number, and are frequently seen in metastatic EC [40]. In addition, *PPP2R1A* and *PPP2R2A* alterations have been associated with whole genome doubling in a pan-cancer study of TCGA data [21]. Further studies are needed to understand the exact role of the PP2A complex in relation to aneuploidy, of particular interest since the PP2A complex might be targetable [39, 41].

Our ‘aneuploidy signature’ based on gene expression data seems to perform equally well as ploidy status estimation by flow cytometry for prediction of aggressive tumors, and its prognostic value was confirmed in an independent TCGA data set (n=338), also in the subgroup of endometrioid tumors. To our knowledge, this is the first study demonstrating a link between an aneuploidy related gene signature, clinicopathologic variables and survival in EC. The score was not associated with survival in subgroup analysis of the non-endometrioid cases, possibly because of the small group size, but potentially also due to more heterogeneous expression patterns in non-endometrioid tumors [36].

Further, the signature revealed an association between aneuploidy and low expression level of chromosome 15q genes, a finding that persisted in subgroup analysis of endometrioid and non-endometrioid tumors by GSEA. TCGA data supported that the observed low expression level of 15q signature genes could be related to altered gene dosage, i.e. deletion. In line with this, Habermann *et al*. identified 15q loss as a frequent phenomenon in aneuploid EC, both within the endometrioid (11% loss) and non-endometrioid (50% loss) subgroups [31]. Also, in the previously mentioned study by Sheffer *et al*, aneuploid colorectal tumors had a high frequency of 15q deletions and correspondingly low 15q gene expression level. Patients with 15q loss also had significantly reduced survival and higher disease stage [35], in line with our findings. Whether this is an aneuploidy associated feature across different tumor types remains to be investigated.

In summary, we have shown that aneuploidy is associated with markers of aggressive endometrial cancer. Aneuploidy independently predicts poor survival adjusted for the commonly applied standard prognostic variables, and identifies patients with high risk of recurrence, lymph node metastasis and poor survival in hormone receptor negative tumors. Thus, aneuploidy should be further explored as a marker to identify patients who could potentially benefit from more aggressive surgical and adjuvant treatment. Aneuploidy associated transcriptional changes point to a complex underlying biological background, and reflects therapeutic challenges in targeting aneuploidy. However, a link towards increased glycolysis in aneuploid tumors is observed, and should be further explored. Our suggested ‘aneuploidy’ signature, linking aneuploidy with low expression of chromosome 15q genes, equally identifies patients with aggressive disease and poor survival, and could provide an alternative method for ploidy status estimation in future diagnostics.

## MATERIALS AND METHODS

### Patient series and tumor samples

The patient population consisted of 1621 women treated for EC at Haukeland University Hospital, Norway, between 1981 and 2015, thoroughly described previously [42]. Approximately half of the patients had flow cytometry assessed ploidy status available, and were included for further analyses (n=825). Data from 363 patients have been published previously [7]. For a subset of the patients, fresh frozen tissue (n=144) and tissue microarrays (TMA, n=526) were available for biomarker studies. Fresh frozen tissue was also available for 30 primary tumors, 18 complex atypical hyperplasias and 42 metastatic lesions without estimated ploidy status [27], used for extended evaluation of our ‘aneuploidy signature’. Estrogen and progesterone receptor (ER/PR) data was available for 1038 patients, 591 with overlapping ploidy status. Clinicopathologic and follow-up data were collected by review of the medical records, and if needed by additional correspondence with the primary physicians and referring hospitals. 92% underwent primary surgical treatment (at least hysterectomy with bilateral salpingo-oophorectomy; HBSO). Details regarding lymphadenectomy routines at different time periods have been described previously [42]. Disease stage was classified according to the FIGO 2009 criteria [43]; cases included prior to 2009 were reclassified. If HBSO was contraindicated, staging was based on curettage results, clinical examination, and preoperative imaging. Follow-up time was defined as the interval between the date of primary treatment and the date of last follow-up/death. Median follow-up time of survivors was 5.02 years (range 0.04 to 23.2); last follow-up was September 1^st^, 2015. The local Institutional Review Board approved the study (IRB-number 2009-2315).

### DNA ploidy analysis

Fresh tissue for DNA ploidy analysis was collected during surgery from an area judged macroscopically representative for the tumor. If HBSO was not performed, ploidy analyses were performed on tissue obtained from palliative surgery (n=7), or curettage (n=6). Tumor tissue was rinsed in phosphate-buffered saline, followed by ethanol fixation. DNA ploidy was analyzed by flow-cytometry, according to previously described protocols [4, 7]. In selected analyses, tetraploid (n=8) and triploid tumors (n=1) were classified together with the aneuploid tumors, as numbers were too low for separate analyses. These nine tumors did not have microarray data and are therefore not included when determining the 9-gene signature described in subsequent sections.

### Expression microarray and data analysis

RNA extracted from fresh frozen tumor tissue was hybridized to Agilent Whole Human Genome Microarray Kit, 44k (catalogue number G4 112F), according to the manufacturers instruction (www.agilent.com) and as described previously [28, 44]. Arrays were scanned using the Agilent Microarray Scanner Bundle. The software J-express (www.molmine.com) [45] was used for microarray analyses. Median spot intensity was used to define the intensity signal, and expression data were quantile normalized and log2-transformed. To identify differentially expressed genes between two groups, significance analysis of microarray (SAM) [46] was performed. For unsupervised hierarchical clustering we used additionally mean scaled expression values, with complete linkage and Pearson correlation as similarity measures.

*Aneuploidy signature:* To identify the minimal set of genes providing the highest discriminatory power between diploid (n=113, 78%) and aneuploid (n=31, 22%) samples, the machine learning method support vector machine (SVM), with 10-fold cross validation, was used on the ranked SAM-list. The method was implemented in R, using the Classification for Microarray package [47]. The genes discriminating between diploid and aneuploid samples with highest accuracy (n=9) were selected. From these genes, a gene signature score was calculated from mean and variance scaled expression values, subtracting the sum of expression values of downregulated genes from the sum of expression values of upregulated genes (in aneuploid compared to diploid samples) [19]. Upper quartile was used as cut-off to separate high and low scores in two groups. One of the identified signature genes; *THC252764* (Agilent probe A_24_P9140887, *RPL4*-variant *Q59GY2*) was not present in the TCGA validation dataset. Since the probe also targets the *RPL4* gene, data for *RPL4* was included in the validation of the signature.

*Connectivity map:* A connectivity map analysis (www.broadinstitute.org/cmap/) [48] was performed to identify compounds generating signatures anti-correlated to the gene list separating aneuploid from diploid tumors. The input signature (SAM, FDR=0 and fold change ±1.5 as cut-off) consisted of 287 differentially expressed genes (204 up- and 83 down-regulated).

*GSEA:* Gene set enrichment analysis (GSEA) (www.broadinstitute.org/gsea) [49], was performed using the Molecular Signatures Database (MSigDB version 5.0, www.broadinstitute.org/gsea/msigdb) datasets c1 (positional gene sets), c2 (curated gene sets), c6 (oncogenic gene sets) and Hallmark gene sets.

### Analyses of the cancer genome atlas (TCGA) data

TCGA endometrial cancer level 3 data for mRNA expression estimated by RNAseq (IlluminaGA_RNAseqV2, downloaded November 20^th^ 2014), and clinical data (downloaded November 3^rd^ 2014, https://tcga-data.nci.nih.gov/tcga) were used for external validation. The ABSOLUTE algorithm was applied to estimate ploidy status for TCGA samples [20]. Cut-off value for diploid tumors was set to 2±0.05; samples with values outside this interval were considered aneuploid. The cBioPortal for cancer genomics [50, 51] and the copy number portal TCGA Tumorscape [21, 22] were applied to assess copy number status for 539 TCGA EC samples without accounting for ploidy status.

### Immunohistochemistry (IHC)

The staining procedure and evaluation for ER and PR on tissue microarrays (TMA) has been described previously [26, 27, 52]. The TMA method has also been described and validated previously [53, 54]. ER and PR status were dichotomized in two categories for binomial analyses: positive ER and/or PR status versus negative ER and PR status by IHC staining. IHC of candidate aneuploidy markers STAG2 and PPP2R3A was performed on TMAs for a subset of 526 and 281 patients, respectively. TMAs were sectioned (5 μm) for immunohistochemical staining: After 20 minutes boiling at pH 9 and peroxidase blocking (S2001 Dako, Denmark), slides were incubated for 60 minutes with STAG2 mouse monoclonal antibody SA-2 (J-12): sc-81852 (Santa Cruz Biotechnology, USA) at 1:500 dilution, or PPP2R3A rabbit polyclonal antibody HPA035829 (Sigma-Aldrich, USA), at 1:100 dilution. Secondary antibody EnVision mouse (labelled polymer-HRP anti-mouse, K4007 Dako, Denmark) was applied for STAG2, and EnVision rabbit (labelled polymer-HRP anti-rabbit, K4003 Dako, Denmark) for PPP2R3A. Dab+Substrate Chromogen System (K3468 Dako, Denmark) was added, and slides counter-stained with hematoxylin. Staining index (SI) was calculated as the product of the area of staining, graded from 0-3, and the intensity of the staining, graded from 0-3, as previously described [55, 56]. For STAG2, nuclear staining was registered, and lower quartile (SI 0-1) defined as low expression. For PPP2R3A, cytoplasmic staining was registered, and upper quartile (SI 6-9) defined as high expression.

### Statistical analyses

Statistical analyses were performed using SPSS (Statistical Package of Social Sciences), version 23.0 (IBM SPSS Statistics, Armonk, NY, USA: IBM Corp, 2015). Associations between categorical variables were assessed by Pearson Chi-square test (Fisher's exact test when appropriate). To compare the distribution of a continuous variable between two or more groups, Mann-Whitney U-test and Kruskal-Wallis test were applied, respectively. Univariate survival analyses were performed by the Kaplan-Meier method, assessing survival differences between groups by the two-sided log-rank test (Mantel-Cox). To determine the optimal cut-off levels for high and low ‘aneuploidy scores’, STAG2 expression level and PPP2R3A expression level, the Kaplan-Meier curves for the variables ranked by tertiles, quartiles and quintiles were examined visually, and the categories showing largest survival differences were chosen. For multivariate survival analyses, the Cox Proportional Hazards Regression Model was used, after visual assessment of included variables by a log-minus-log plot to check the proportional hazards assumption. For the survival analyses, disease specific survival (DSS) was defined as primary endpoint, except in TCGA data, where overall survival (OS) was used. Binary logistic regression analysis was performed for prediction of recurrence and lymph node metastasis. A p-value below 0.05 was set as threshold significance level for all the statistical analyses.

The authors would like to thank Bendik Nordanger, Britt Edvardsen, Ellen Valen, Gerd Lillian Halseth, Hua My Hoang, Kadri Madissoo and Reidun Kopperud for technical assistance.

## SUPPLEMENTARY MATERIALS FIGURES AND TABLES






