# Supplementary material for: Aneuploidy related transcriptional changes in endometrial cancer link low expression of chromosome 15q genes to poor survival
Source: Oncotarget. 2016 Dec 25;8(6):9696–707. doi: 10.18632/oncotarget.14201 (PMC5354764; doi:10.18632/oncotarget.14201)
Supplement: Supplementary file 1 [file oncotarget-08-9696-s001.pdf]

Aneuploidy related transcriptional changes in endometrial cancer link low expression of chromosome 15q genes to poor survival

SUPPLEMENTARY FIGURES AND TABLES

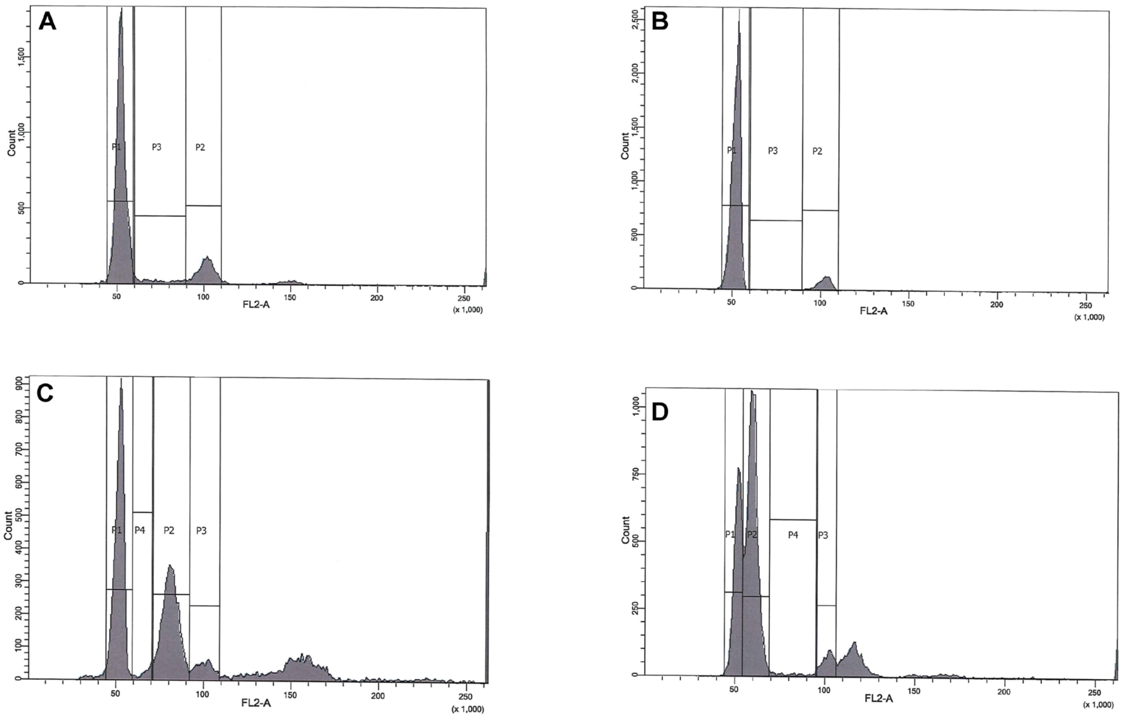

Supplementary Figure 1: DNA histograms from diploid and aneuploid tumors. Example histograms from flow cytometric assessment of DNA ploidy status. X-axis: emitted fluorescent light from labeled DNA, Y-axis: cell count. Panels **A** and **B.** Histograms from diploid tumors, showing a major peak (P1) representing cells with a diploid DNA content, and a minor peak (P2) representing cells in M-phase of cell cycle. Panels **C** and **D.** Histograms from aneuploid tumors, showing multiple peaks representative for cell subpopulations with a non-diploid DNA content.

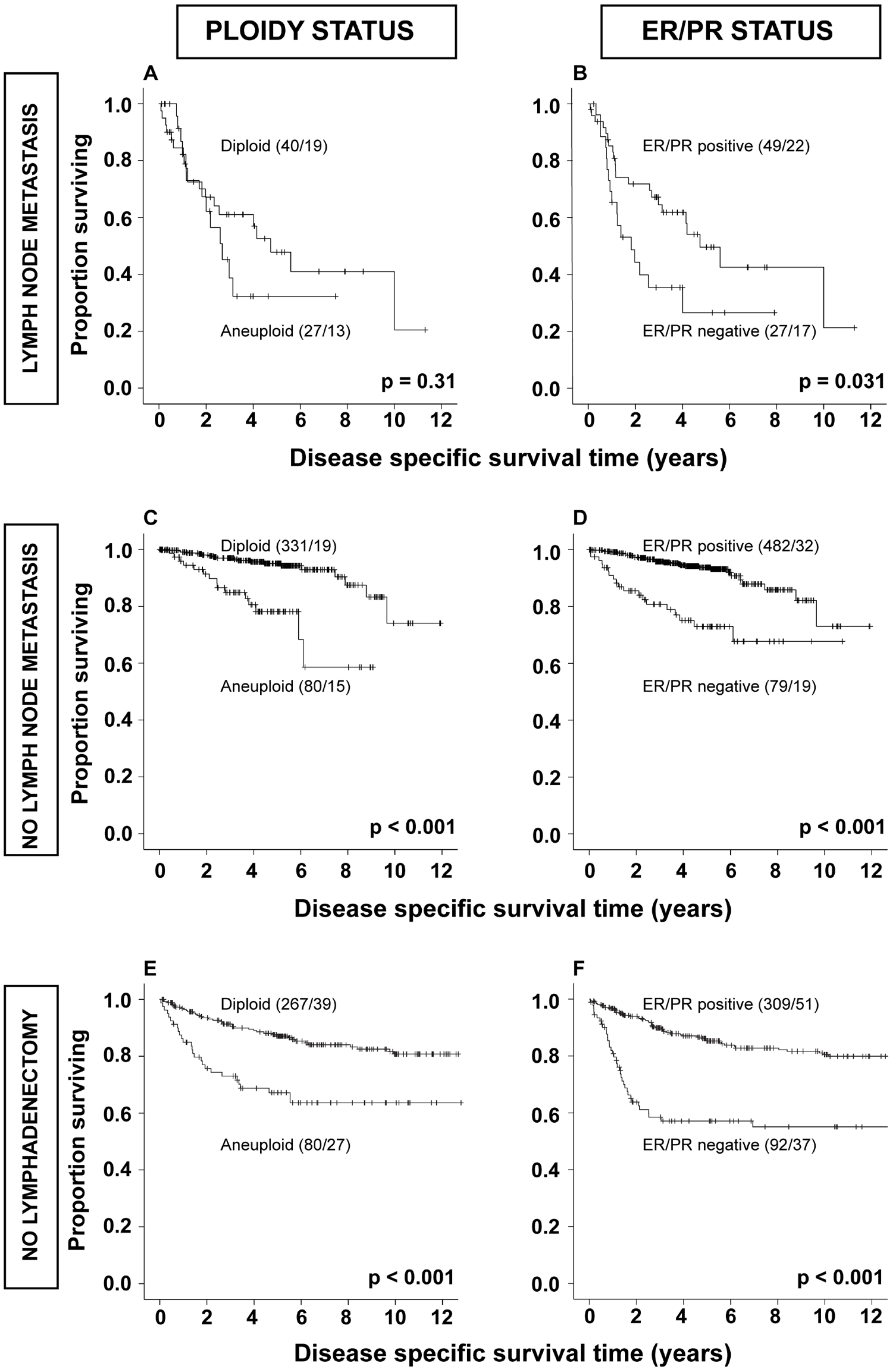

Supplementary Figure 2: Comparison of prognostic value of ploidy and ER/PR status. Survival according to flow cytometry assessed ploidy status compared to ER/PR status in patients with lymph node metastasis (upper panel), no lymph node metastasis (mid panel) and no lymphadenectomy (uncertain lymph node status, lower panel). **A, C** and **E.** Diploid versus aneuploid tumors. **B, D** and **F.** ER and/or PR positive versus ER and PR negative status.

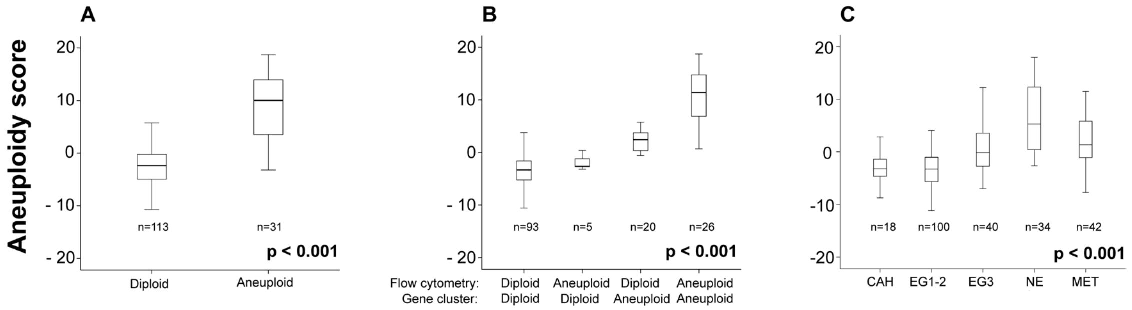

Supplementary Figure 3: 'Aneuploidy signature' score distribution. 'Aneuploidy signature' score for diploid and aneuploid tumors by flow cytometry **A.** four groups based on ploidy status by flow cytometry and cluster formation: concordant diploid/aneuploid versus discordant diploid/aneuploid status **B.** and complex atypical hyperplasia (CAH), endometrioid tumors grade 1-2 (EG1-2), endometrioid tumors grade 3 (EG3), non-endometrioid tumors (NE) and metastases (MET) **C.** n=number of patients in each group. P-values represent Mann Whitney (A) and Kruskall Wallis (B and C) tests for significance of differences between groups.

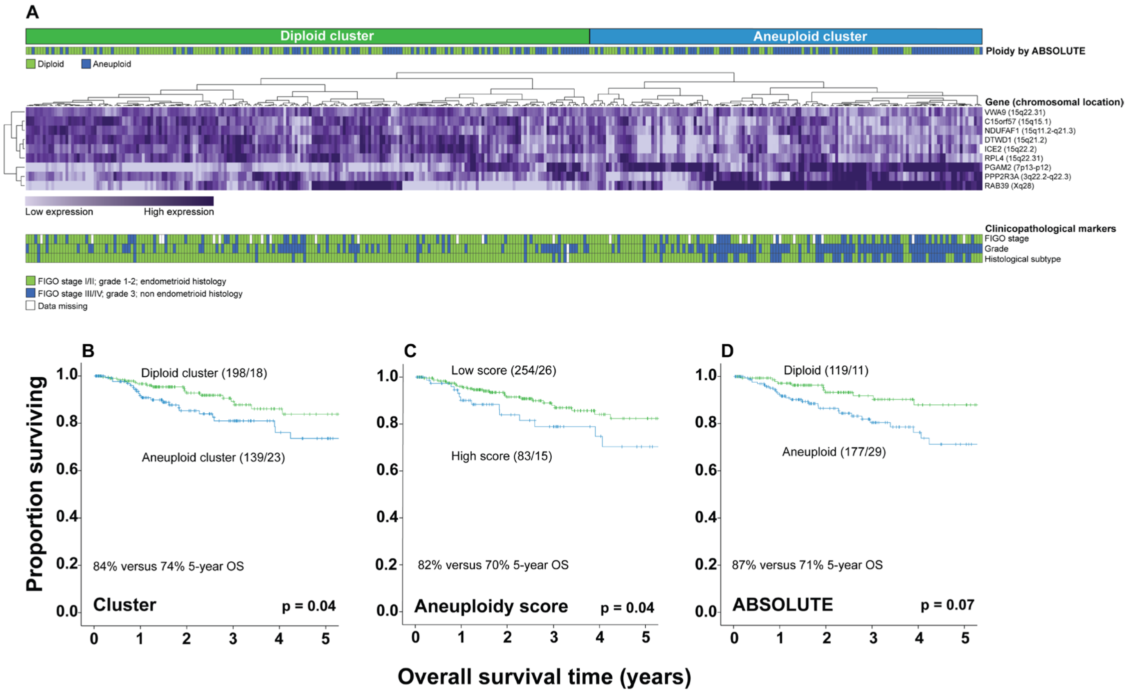

Supplementary Figure 4: 'Aneuploidy signature' validation in TCGA data. Cluster formation based on 'aneuploidy signature' related to ploidy status estimated by ABSOLUTE (Carter et al, 2012), FIGO stage, grade and histological subtype in a validation data set of 338 TCGA EC samples. Unsupervised hierarchical clustering reveals a similar pattern as demonstrated in Figure 2 **A.** OS for patients segregating within the 'diploid' compared to the 'aneuploid cluster' **B.** for patients with low compared to high 'aneuploidy score' **C.** and for patients with diploid compared to aneuploid tumors by ABSOLUTE **D.**

A

	This study data set (n=144)			TCGA data set (n=337)	
	Endometrioid n=116	Non-endometrioid n=28		Endometrioid n=272	Non-endometrioid n=65
	0.01	0.03	**FIGO stage** (I/II vs III/IV)	0.69	0.37
	<0.001		**Histologic grade** (grade1-2 vs grade3)	<0.001	
	0.01	0.38	**Age** (below vs above median, 66.2 years)	0.01	0.06
	<0.001	<0.001	**Ploidy status** (diploid vs aneuploid by flow)	<0.001	0.05

Higher aneuploidy score in FIGO stage III/IV vs I/II; grade 3 vs grade 1-2 tumors; age above median vs below median; and aneuploid vs diploid tumors
Higher aneuploidy score in FIGO stage I/II vs III/IV
Non-endometrioid tumors = grade 3

B

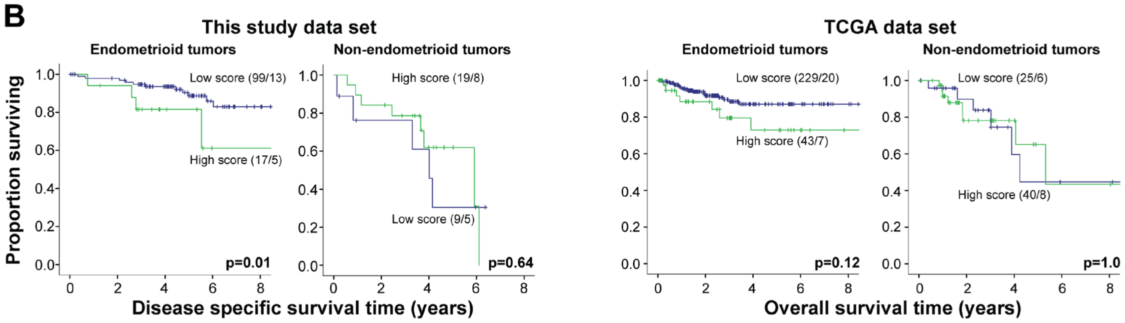

C

Enriched in endometrioid diploid tumors

Rank	Gene set	Size	p-value	FDR (%)
3	**chr15q22**	71	0	0.11
4	**chr15q21**	73	0	0.11
7	**chr15q11**	27	0	0.74
9	**chr15q14**	37	0	1.8
12	**chr15q15**	54	0	3.15
18	**chr15q13**	30	0	4.35
25	**chr15q24**	58	0.01	6.55
26	**chr15q25**	36	0.01	6.35
30	**chr15q23**	28	0.02	7.88

Enriched in non-endometrioid diploid tumors

Rank	Gene set	Size	p-value	FDR (%)
1	**chr15q21**	73	0	0
2	**chr15q14**	37	0	0
3	**chr15q22**	71	0	0.1
6	**chr15q26**	51	0	2.28
9	**chr15q24**	58	0	2.78
15	**chr15q15**	54	0	4.96
16	**chr15q25**	36	0	5.15
41	**chr15q23**	28	0.04	16.97

Supplementary Figure 5: Summary of subgroup analysis of 'aneuploidy signature' in endometrioid and non-endometrioid tumors. Top panel **A.** represents heatmap of p-values from assessment of differential distribution of 'aneuploidy score' according to clinicopathologic variables in this study data set and TCGA data (Mann-Whitney U test). n=number of patients in each category. P-values are colored according to whether the score shows similar distribution pattern in subgroup analysis as when all histologic subtypes were examined together (green); or opposite distribution pattern (red). Mid panel **B.** shows survival according to aneuploidy score for endometrioid and non-endometrioid tumors separately, in this study data set and in TCGA data. Lower panel **C.** shows results from GSEA analyses on this study data set. Chromosome 15q related gene sets with p<0.05 and FDR<25% are represented.

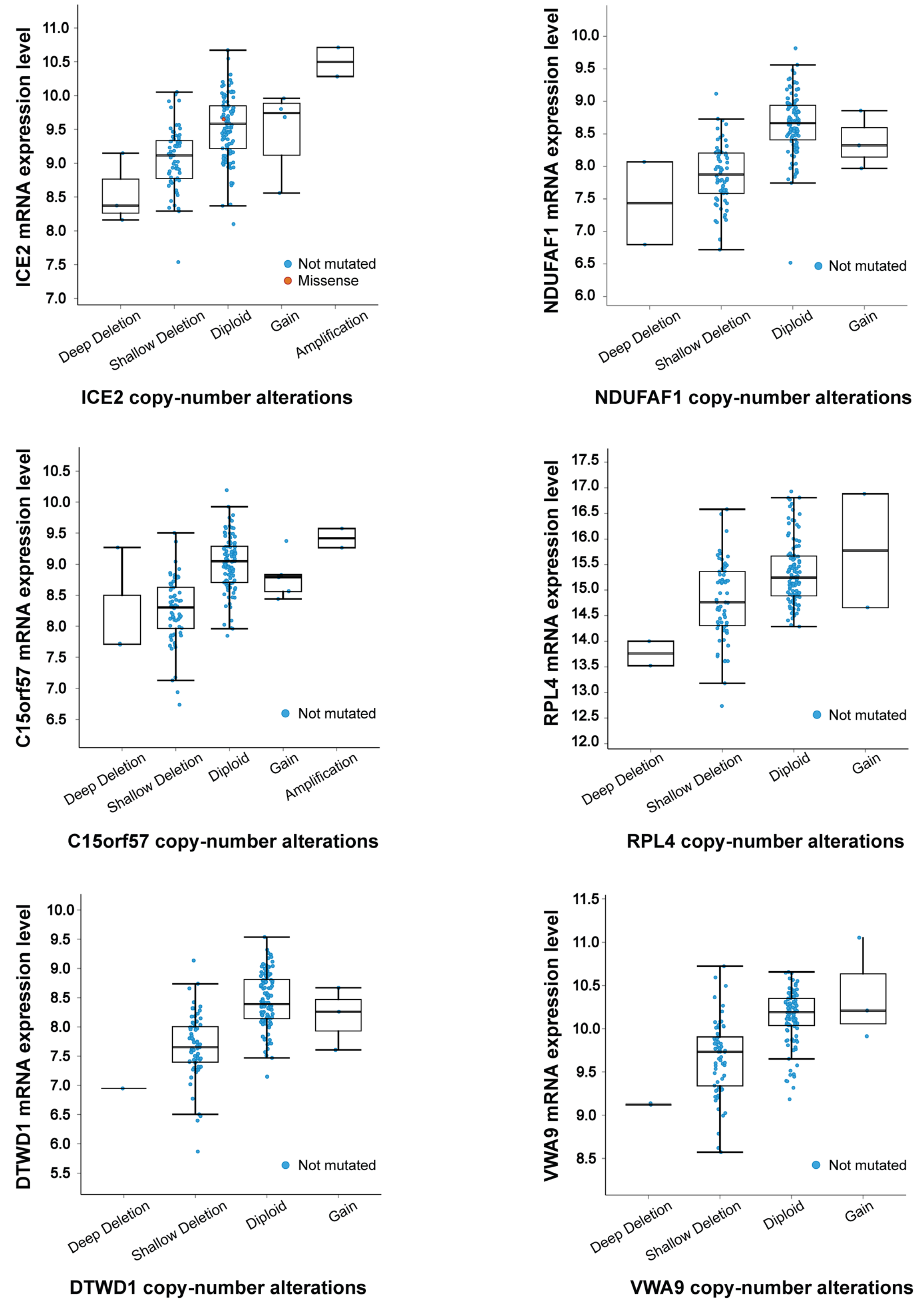

Supplementary Figure 6: mRNA expression level and corresponding copy number for chromosome 15q genes. mRNA expression level in relation to copy number analysis of the six downregulated 'aneuploidy signature' genes for 539 TCGA EC samples. 'Deep deletion' indicates possibly homozygous deletion, 'Shallow deletion' indicates possibly heterozygous deletion. Likewise, 'Gain' indicates low level gain, 'Amplification' indicates high level amplification, as determined by the GISTIC algorithm (Beroukhim et al, 2007). Figure adapted from www.cbioportal.org (Cerami *et al*, 2012; Gao *et al*, 2013).

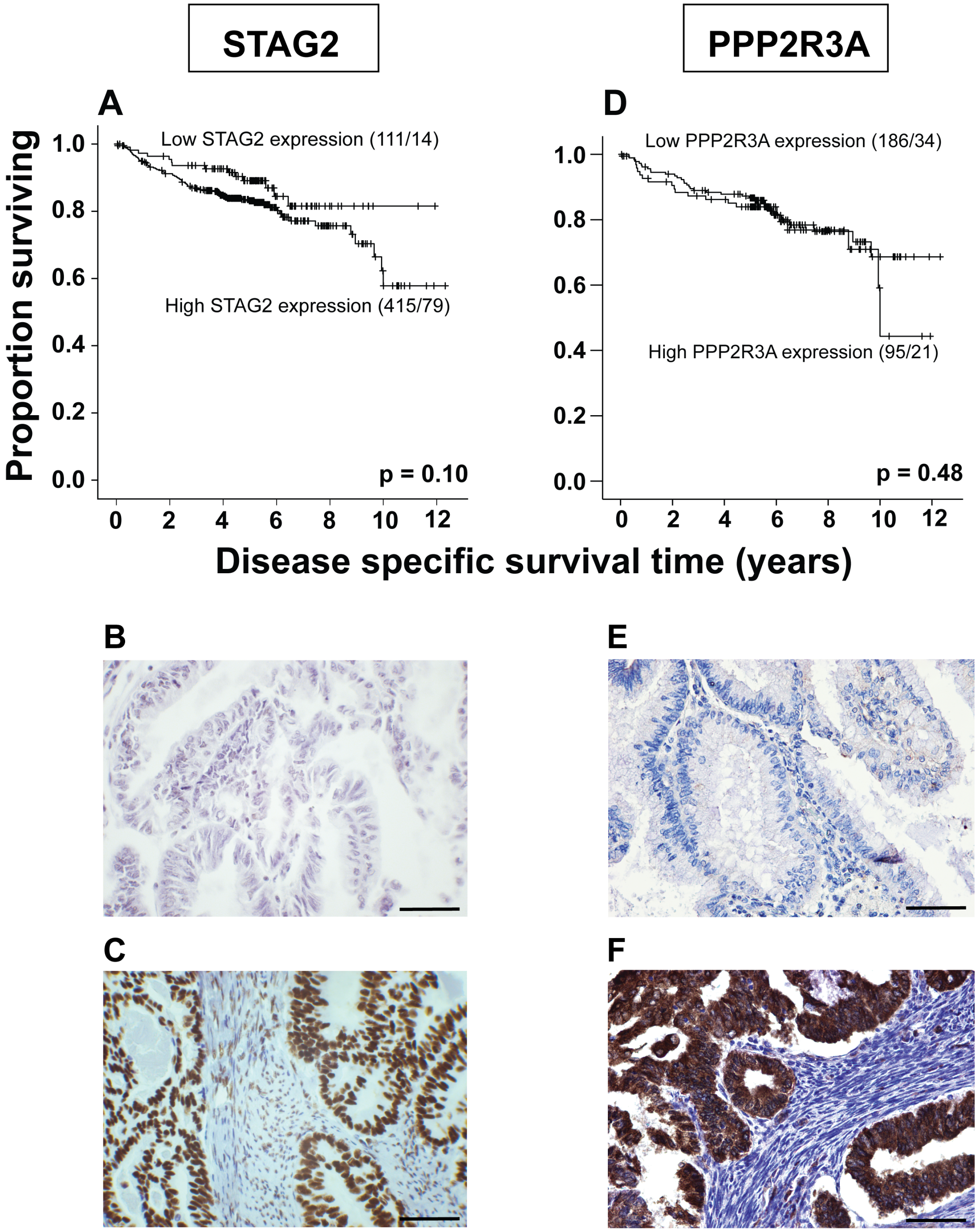

Supplementary Figure 7: Immunohistochemical assessment of suggested candidate aneuploidy markers STAG2 and PPP2R3A. Top left panel **A.** illustrates no survival difference according to STAG2 expression by IHC for 526 EC patients. Representative staining for low STAG2 expression (SI 0) **B.** and high STAG2 expression (SI 9) **C.** are shown. Top right panel **D.** illustrates no survival difference according to PPP2R3A expression by IHC for 281 EC patients. Representative staining for low PPP2R3A expression (SI 0) **E.** and high PPP2R3A expression (SI 9) **F.** are shown. Scale bar: 40 µm (B-C; E-F).

Supplementary Table 1: Prognostic impact of ploidy status and ER/PR status adjusted for standard clinicopathologic variables (Cox regression model)

A. Survival analysis according to standard clinicopathologic variables and ploidy status by flow cytometry (n=804)

	Patients, n (%)	Unadjusted HR	95% CI	p-value	Adjusted HR	95% CI	p-value
**Age**	804 (100)	1.05	1.04 - 1.07	<0.001	1.04	1.02 - 1.06	<0.001
**Histologic type and grade**				<0.001			<0.001
Endometrioid grade 1-2	538 (67)						
Endometrioid grade 3	119 (15)	3.48	2.20 - 5.50		2.17	1.35 - 3.49	
Non-endometrioid	147 (18)	7.04	4.71 - 10.51		3.06	1.95 - 4.81	
**FIGO stage**				<0.001			<0.001
Stage I + II	677 (84)						
Stage III + IV	127 (16)	9.46	6.67 - 13.42		7.10	4.90 - 10.27	
**Ploidy status**				<0.001			0.013
Diploid	617 (77)						
Aneuploid	187 (23)	2.91	2.05 - 4.13		1.62	1.11 - 2.37	

B. Survival analysis according to standard clinicopathologic variables and ER/PR status (n=1025)

	Patients, n (%)	Unadjusted HR	95% CI	p-value	Adjusted HR	95% CI	p-value
**Age**	1025 (100)	1.05	1.04 – 1.06	<0.001	1.04	1.02 – 1.05	<0.001
**Histologic type and grade**				<0.001			<0.001
Endometrioid grade 1-2	711 (69)						
Endometrioid grade 3	142 (14)	2.93	1.95 – 4.38		1.73	1.14 – 2.63	
Non-endometrioid	172 (17)	6.69	4.79 – 9.35		2.76	1.86 – 4.09	
**FIGO stage**				<0.001			<0.001
Stage I + II	856 (84)						
Stage III + IV	169 (16)	9.18	6.82 – 12.38		6.35	4.63 – 8.72	
**ER/PR status**				<0.001			0.005
ER and/or PR positive	828 (81)						
ER and PR negative	197 (19)	3.77	2.79 – 5.08		1.63	1.16 – 2.29	

n=number of patients in each category; HR: Hasard Ratio; CI: Confidence Interval

Supplementary Table 2: Binary logistic regression models for prediction of recurrence and lymph node metasasis

A. Prediction of recurrence for patients with ER/PR positive versus ER/PR negative tumors (n=542)

	n	Unadjusted OR	95% CI	p-value	Adjusted OR	95% CI	p-value
**ER and/or PR positive tumors**	446						
**Histologic type and grade**				<0.001			<0.001
Endometrioid grade 1-2	343						
Endometrioid grade 3	57	2.43	1.24 – 4.74		2.38	1.20 – 4.70	
Non-endometrioid	46	5.71	2.95 – 11.05		5.51	2.74 – 11.06	
**Ploidy status**				0.039			0.755
Diploid	370						
Aneuploid	76	1.85	1.03 – 3.30		1.11	0.58 – 2.12	
**ER and PR negative tumors**	96						
**Histologic type and grade**				0.017			0.092
Endometrioid grade 1-2	26						
Endometrioid grade 3	24	0.67	0.16 – 2.73		0.53	0.12 – 2.34	
Non-endometrioid	46	3.06	1.04 – 9.00		2.1	0.64 – 6.54	
**Ploidy status**				<0.001			0.002
Diploid	61						
Aneuploid	35	5.44	2.17 – 13.66		4.67	1.78 – 12.27	

B. Prediction of lymph node metastasis for patients with ER/PR positive versus ER/PR negative tumors (n=415)

	n	Unadjusted OR	95% CI	p-value	Adjusted OR	95% CI	p-value
**ER and/or PR positive tumors**	339						
**Histologic type and grade**				<0.001			<0.001
Endometrioid grade 1-2	245						
Endometrioid grade 3	46	4.72	1.86 – 11.99		4.47	1.71 – 11.68	
Non-endometrioid	48	7.21	3.05 – 17.07		6.71	2.69 – 16.72	
**Ploidy status**				0.022			0.635
Diploid	279						
Aneuploid	60	2.50	1.14 – 5.46		1.23	0.52 – 2.92	
**ER and PR negative tumors**	76						
**Histologic type and grade**				0.680			0.236
Endometrioid grade 1-2	12						
Endometrioid grade 3	18	0.75	0.15 – 3.75		0.59	0.11 – 3.23	
Non-endometrioid	46	0.56	0.14 – 2.23		0.27	0.05 – 1.34	
**Ploidy status**				0.018			0.007
Diploid	46						
Aneuploid	30	3.71	1.25 – 11.01		5.47	1.58 – 18.99	

n=number of patients in each category; OR: Odds Ratio; CI: Confidence Interval

Supplementary Table 3: Significance analysis of microarray (SAM) of 113 diploid versus 31 aneuploid endometrial tumors. Genes with FDR=0 and q-value <0.05 are listed

See Supplementary File 1

Supplementary Table 4: Distribution of 'aneuploidy score' for 144 endometrial cancer patients according to standard clinicopathologic variables

	Patients, n (%)	Median score	p-value*
**FIGO stage**			0.007
Stage I-II	115 (80)	-2.3	
Stage III-IV	29 (20)	0.2	
**Histologic type and grade**[a]			<0.001
Endometrioid grade 1-2	84 (59)	-3.4	
Endometrioid grade 3	31 (22)	0.2	
Non-endometrioid	28 (19)	6.7	
**Age (median)**			<0.001
< 66.2 years	65 (45)	-2.7	
≥ 66.2 years	79 (55)	-0.2	
**Ploidy status (by flow cytometry)**			<0.001
Diploid	113 (78)	-2.4	
Aneuploid	31 (22)	10.0	
**ER/PR status**[b]			<0.001
ER and/or PR positive	115 (83)	-2.3	
ER and PR negative	23 (17)	2.5	

n=number of patients in each catgory; *: Mann-Whitney U-test for two categories, Kruskal-Wallis test for three categories; [a]: Data missing for 1 patient; [b]: Data missing for 6 patients

Supplementary Table 5: Gene set enrichment analysis for diploid (n=113) versus aneuploid (n=31) tumors Gene sets with FDR<25% and p-value < 0.05 are listed

See Supplementary File 2

Supplementary Table 6: Drug signatures identified by Connectivity map as negatively correlated with gene list separating diploid and aneuploid samples (SAM, FDR=0, Fold Change ± 1.5)

Rank	Drug name	n	Enrichment score	p-value	Drug target/action
**1**	Adiphenine	5	-0.82	0.00046	Inhibitor of nicotinic receptors
**2**	Isoflupredone	3	-0.929	0.00052	Glucocorticoid
**3**	Nadolol	4	-0.866	0.00062	Beta-blocker
**4**	Colistin	4	-0.843	0.00105	Polymyxin antibiotic
**5**	Haloperidol	32	-0.327	0.00143	Antipsychotic
**6**	Geldanamycin	15	-0.464	0.00184	Hsp90 inhibitor
**7**	Viomycin	4	-0.821	0.00195	Non-ribosomal peptide antibiotic
**8**	Wortmannin	18	-0.413	0.00308	PI3Kinase inhibitor
**9**	Genistein	17	-0.417	0.0036	Phytoestrogen, isoflavone
**10**	Maprotiline	4	-0.785	0.00426	Tetracyclic antidepressant
**11**	Levomepromazine	4	-0.78	0.00487	Phenotiazine, neuroleptic
**12**	Midodrine	5	-0.701	0.00519	Alpha1-receptor agonist
**13**	Dihydroergocristine	4	-0.769	0.00573	Ergot alkaloid
**14**	Carbimazole	3	-0.855	0.00603	Thyroid peroxidase inhibitor
**15**	Felbinac	4	-0.751	0.00774	NSAID

n=number of times the compound was tested in the Connectivity Map.

Supplementary Table 7: STAG2 and PPP2R3A expression estimated by immunohistochemistry (staining index, SI) in relation to standard clinicopathologic variables

	STAG2 expression			PPP2R3A expression		
	Low, n (%)	High, n (%)	p-value*	Low, n (%)	High, n (%)	p-value*
**Age, quartiles**			0.71			0.58
<58	29 (22)	102 (78)		45 (63)	27 (38)	
58 – 66	33 (23)	108 (77)		51 (65)	27 (35)	
66 – 75	27 (21)	103 (79)		39 (64)	22 (36)	
≥ 75	22 (18)	102 (82)		51 (73)	19 (27)	
**Histologic subtype and grade**			0.15			0.72
Endometrioid grade 1-2	82 (23)	271 (77)		129 (66)	68 (35)	
Endometrioid grade 3	14 (20)	57 (80)		25 (62)	15 (38)	
Non-endometrioid	14 (14)	84 (86)		29 (71)	12 (29)	
**FIGO stage**			0.87			0.86
Stage I	85 (21)	318 (79)		149 (67)	72 (33)	
Stage II	9 (25)	27 (75)		11 (61)	7 (39)	
Stage III	13 (21)	49 (79)		17 (63)	10 (37)	
Stage IV	4 (16)	21 (84)		9 (60)	6 (40)	
**Ploidy status (flow cytometry)**			0.43			0.95
Diploid	68 (24)	211 (76)		111 (70)	47 (30)	
Aneuploid	15 (20)	60 (80)		23 (70)	10 (30)	
**ER status**			0.01			0.80
ER positive	74 (19)	322 (81)		138 (66)	70 (34)	
ER negative	37 (30)	87 (70)		44 (65)	24 (35)	
**PR status**			0.08			0.32
PR positive	89 (23)	304 (77)		131 (65)	70 (35)	
PR negative	20 (16)	109 (85)		55 (71)	22 (29)	

n=number of patients in each category; *: Pearson χ^2-test
